# Unusual backfolded binding poses of BAZ2A bromodomain binders

**DOI:** 10.1107/S2059798326004596

**Published:** 2026-06-03

**Authors:** Andrea Dalle Vedove, Giulia Cazzanelli, Vito Giuseppe D’Agostino, Pablo Andres Vargas Rosales, Amedeo Caflisch, Graziano Lolli

**Affiliations:** a Department of Cellular, Computational and Integrative Biology – CIBIO University of Trento Via Sommarive 9 38123Povo – Trento Italy; b Department of Biochemistry University of Zurich Winterthurerstrasse 190 CH-8057Zurich Switzerland; National Hellenic Research Foundation, Greece

**Keywords:** BAZ2A, bromodomains, X-ray crystallography, small molecules, protein–protein interactions

## Abstract

BAZ2A is a large multidomain protein overexpressed in aggressive prostate cancer, where it potentiates migration and invasion of other tissues. To counteract its metastasis-promoting role, small molecules interfering with the recognition of acetylated lysines by the BAZ2A bromodomain have been identified. However, unlike other bromodomains, BAZ2A has a shallow pocket, which significantly complicates the development of potent inhibitors. Here, we report the exploration of the acetyl-pyrrole scaffold, leading to the identification of BAZ2A-binding compounds assuming a peculiar, almost enclosed, conformation, as determined by X-ray crystallography. These molecules pose the basis for the development of potent BAZ2A macrocyclic inhibitors, as performed for other bromodomains.

## Abstract 2

We identified BAZ2A-binding compounds assuming a peculiar, almost enclosed, conformation. These molecules pose a basis for the development of potent BAZ2A macrocyclic inhibitors, as performed for other bromodomains.

## 1. Introduction

Epigenetic modulation through bromodomain inhibitors is a therapeutic strategy currently being explored for the treatment of various malignancies and inflammatory diseases (Zaware & Zhou, 2019 ▸; Cochran *et al.*, 2019 ▸). Different acetyl-lysine (Kac)-mimicking scaffolds have been explored, with potent inhibitors having been developed and successfully advanced to the clinic, particularly for the BET (bromo­domain and extra-terminal domain) subfamily (Bechter & Schöffski, 2020 ▸; Guo *et al.*, 2023 ▸. Other bromodomains proved to be more challenging to target due to their shallow binding pockets and limited opportunities for high-affinity ligand engagement.

A variety of heteroaromatic chemotypes have been explored as Kac mimetics, including triazoles, isoxazoles, quinazolinones, and acyl- and acetyl-pyrrole derivatives. The latter two have emerged as versatile KAc-mimicking scaffolds capable of engaging the conserved bromodomain cavity. Across different bromodomain subfamilies, acyl-pyrrole-based ligands have shown variable levels of potency: in BET, BRD7/9 and CREBBP bromodomains, optimized compounds have reached submicromolar affinities, while acetyl-pyrrole-containing ligands targeting the BRPF1 bromodomain have been reported in the single-digit micromolar range (Xu *et al.*, 2016 ▸; Hügle *et al.*, 2016 ▸, 2017 ▸, 2020 ▸; Lucas *et al.*, 2013 ▸; Cazzanelli *et al.*, 2024 ▸).

The bromodomain present in BAZ2A (bromodomain adjacent to zinc-finger domain protein 2A) is among the most difficult to target (Cazzanelli *et al.*, 2023 ▸). BAZ2A is a large multidomain protein part of the NoRC (nucleolar remodeling complex) involved in silencing of rDNA (Zhou & Grummt, 2005 ▸). BAZ2A is, however, overexpressed in aggressive and recurrent prostate cancer, where it cooperates with EZH2 (enhancer of zeste homolog 2) and potentiates the migration and metastatic potential of tumor cells (Gu *et al.*, 2015 ▸). Despite its biological relevance, the development of potent and selective BAZ2A inhibitors remains difficult. The most advanced chemical probes reported to date, such as GSK2801 and BAZ2-ICR, display submicromolar affinities (IC_50_ values of 0.40 and 0.13 µ*M*, respectively), highlighting the inherent challenges associated with this target (Chen *et al.*, 2016 ▸; Drouin *et al.*, 2015 ▸). Moreover, fragment-based screening approaches have shown that initial BAZ2A binders often exhibit weak affinities in the high-micromolar range, requiring extensive optimization to reach single-digit micromolar potency (Dalle Vedove *et al.*, 2022 ▸).

We recently identified a 3-acetyl-4-ethyl-2-methyl-5-thiazole-pyrrole fragment as a promising scaffold for growing BAZ2A binders with affinity in the single-digit micromolar range (Dalle Vedove *et al.*, 2022 ▸). The BAZ2A affinity of the initial fragment could be increased by adding substituents able to interact with the Glu1820 side-chain and/or Asn1823 main-chain nitrogen. These interactions with the rim of the binding pocket reinforce those formed by the headgroup with the pocket core, also providing selectivity determinants (Cazzanelli *et al.*, 2024 ▸; Dalle Vedove *et al.*, 2021 ▸).

Building on these findings, we report here on additional acetyl-pyrrole BAZ2A binders identified through a pharmacophore interrogation of the commercially available chemical space and confirmed by a competitive binding assay and X-ray crystallography. While the binding affinities of the compounds described here remain in the micromolar range, they are consistent with early-stage BAZ2A ligands and reflect the intrinsic difficulty of this target.

## 2. Experimental procedures

Compounds were selected at https://pharmit.csb.pitt.edu (Sunseri & Koes, 2016 ▸) as described above and were purchased from either Enamine Ltd or SIA Chemspace with a purity of >95%.

Recombinant His-tagged BAZ2A bromodomain was tested by Alpha technology in the presence of a biotinylated histone H3 acetylated lysine 14 peptide [H3K(Ac)14, H-YQTARKSTGGK(Ac)APRKQLATKAK(Biotin)-OH] using the AlphaScreen histidine-detection kit (PerkinElmer). The assays were performed in 384-well OptiPlates (PerkinElmer) using an equimolar amount of peptide at the hooking point (750 nm) evaluated after incubation for 1 h at room temperature in a solution consisting of 50 m*M* HEPES pH 7.4, 100 m*M* NaCl, 0.1% BSA, 0.05% CHAPS. All compounds were tested in duplicate at 100 µ*M*. Compound **28** was tested in dose–response and an IC_50_ value was obtained by nonlinear regression of the log(dose)–response fit using the *GraphPad Prism* software.

BAZ2A bromodomain was purified as reported in Spiliotopoulos *et al.* (2017 ▸). BAZ2A was co-crystallized with the compounds of interest (5 m*M* or a saturating solution for less soluble compounds) by microseeding of apo BAZ2A crystals at 4°C in a solution consisting of Tris pH 8, 0.2 *M* MgCl_2_, 20% PEG 3350. DMSO was avoided or kept to a minimum (0.1%) in the co-crystallization experiments as it can compete for binding to the bromodomain Kac pocket (Lolli & Battistutta, 2013 ▸; Marchand *et al.*, 2016 ▸, 2017 ▸). Co-crystals were cryoprotected with ethylene glycol and flash-cooled in liquid nitrogen.

Diffraction data were collected on the XRD2 beamline of the Elettra Synchrotron Light Source, Trieste, Italy. Data processing was performed using *XDS* (Kabsch, 2010 ▸), followed by scaling with *AIMLESS* (Evans & Murshudov, 2013 ▸; Agirre *et al.*, 2023 ▸). The high-resolution cutoff was determined according to the criteria of Karplus & Diederichs (2015 ▸).

**Figure 1:**
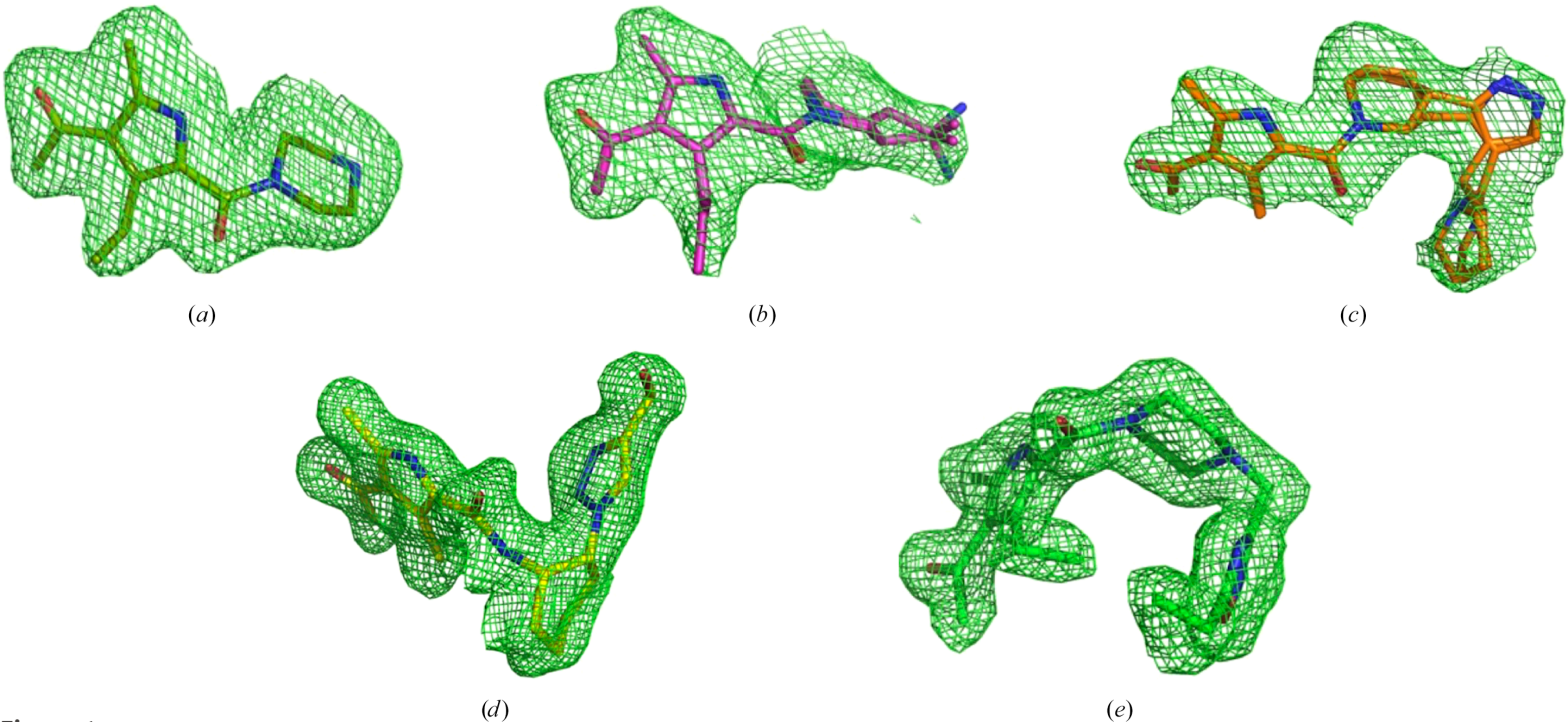
*F*_o_ − *F*_c_ polder OMIT maps contoured at 3σ for compounds **1** (*a*), **23** (*b*), **26** (*c*), **27** (*d*) and **28** (*e*).

Structures were solved by molecular replacement using *Phaser* (Agirre *et al.*, 2023 ▸; McCoy *et al.*, 2007 ▸) with PDB entry 5mgj (Spiliotopoulos *et al.*, 2017 ▸). Initial models were refined through iterative cycles of automated refinement using *Phenix* (Liebschner *et al.*, 2019 ▸), combined with manual model building in *Coot* (Emsley *et al.*, 2010 ▸). X-ray data-collection and refinement statistics are reported in Table 1 ▸. *F*_o_ − *F*_c_ electron-density maps for compounds in complex with the BAZ2A bromodomain are shown in Fig. 1 ▸.

BAZ2A structures were deposited in the PDB as entries 9f6w (compound **1**), 9f70 (compound **23**), 9f71 (compound **26**), 9f77 (compound **27**) and 9f78 (compound **28**).

## 3. Results and discussion

A *Pharmit* (Sunseri & Koes, 2016 ▸) search was performed by spatially imposing all chemical features of a 3-acetyl-2,4-dimethyl-pyrrole headgroup (optimal interactions at the bottom of the pocket) and a positive-charge or hydrogen-bond donor for interaction with Glu1820 at the pocket tip and/or a hydrogen-bond acceptor for interaction with the proximal Asn1823 (Supplementary Fig. S1). The search returned 404 compounds, which were visually inspected: about 65% clashed with the protein chain, or showed protruding solvent-exposed regions or charged groups (especially nitro groups) inside the pocket cavity, and were excluded. We focused on 3-acetyl-4-alkyl-2-methyl-5-carboxamide-pyrrole-containing compounds, which were by far the most represented in the *Pharmit* selection. 28 compounds were readily available from commercial sources and were tested for their ability to interfere with BAZ2A binding to an acetylated peptide (Table 2 ▸). Seven compounds showed >50% reduction of BAZ2A binding when tested at 100 µ*M*. Crystallographic structures in complex with the BAZ2A bromodomain were solved for four of them (compounds **23**, **26**, **27** and **28**), and for compound **1**, the prototype fragment for the set, while for the remaining three compounds (**15**, **16** and **25**) crystals in complex with BAZ2A did not improve over very thin and poorly diffracting needles.

**Figure 2:**
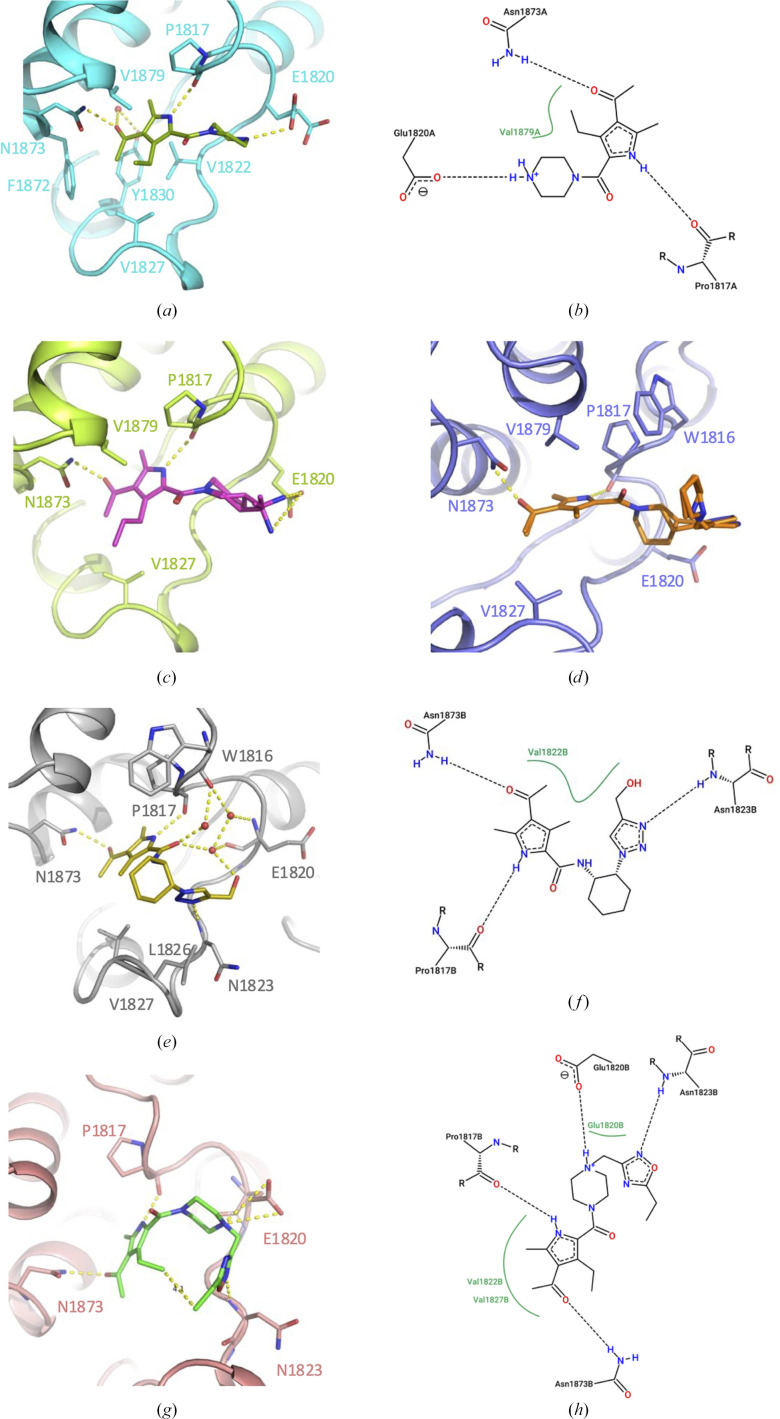
Crystallographic structures of compounds **1** (*a*), **23** (*c*), **26** (*d*), **27** (*e*) and **28** (*g*). Detailed interactions with the BAZ2A bromodomain are depicted for compounds **1** (*b*), **27** (*f*) and **28** (*h*) and were obtained with *PoseView* (Stierand & Rarey, 2010 ▸). For compounds **27** and **28**, only chain *B* is shown; the compounds assume very similar poses in all chains; see also Table 3 ▸ and Supplementary Fig. S3.

As expected, the pyrrole ring in compound **1** is sandwiched between the Val1822 and Val1879 side chains and forms a hydrogen bond with the Pro1817 main-chain oxygen (Figs. 2 ▸*a* and 2 ▸*b*). The 2-methyl group is nicely located in the small hydrophobic cavity occupied by the same group of the natural Kac ligand. At the same time, the 4-ethyl substituent provides additional van der Waals interactions with Val1827 and Val1879. The acetyl group is involved in two hydrogen bonds with the Asn1873 and Tyr1830 side chains (the latter being water-mediated), further contacting the side chains of Val1827 and Phe1872. The piperazine ring forms a salt bridge with Glu1820; however, the electron density for this residue is diffuse, suggesting flexibility and a suboptimal interaction.

The above-described interactions for the substituted acetyl-pyrrole ring are conserved in all other compounds, with differences arising in the tail region. The interaction of the 3-(1-aminoethyl)piperidine with Glu1820, here with well defined electronic density, appears ameliorated in compound **23** (Fig. 2 ▸*c*), which was tested as a mixture of four stereoisomers both for crystallization and in the biochemical assay. The electron density suggests that all of them bind, but placing the amino group at different distances from the Glu1820 side chain (3.5 to 5.2 Å), with a possible effect on their relative affinities for the target. Conversely, the pyrrolidine-pyrazol-piperidine tail of compound **26** is only in van der Waals contact with the Trp1816 and Glu1820 side chains, being otherwise exposed to the solvent (Fig. 2 ▸*d*). The tail of compound **27** takes a different direction starting from the amide carbonyl, here rotated by about 130° with respect to the parent compound **1** and involved in a water-bridged hydrogen bond with the Trp1816 main-chain oxygen (Figs. 2 ▸*e* and 2 ▸*f*). Its cyclohexyl group is partially in hydrophobic contact with Leu1826 and Val1827, while the triazole ring forms a hydrogen bond with the Asn1823 main-chain nitrogen. The terminal hydroxyl, however, fails to contact Glu1820. Interestingly, a water molecule bridges the terminal hydroxyl with the amide carbonyl, suggesting cyclization as a possible development for this compound. Tetrahedral coordination for the same water molecule is completed by hydrogen bonds to the Glu1820 main-chain oxygen and an additional water molecule strongly connected to the protein backbone. The BAZ2A–compound **27** complex crystallized in a different space group (*P*2_1_2_1_2_1_) to the canonical *P*3_1_21 observed with compounds **1**, **23** and **26**. The new crystal packing is dictated by a stacking interaction between the cyclohexyl-triazole tail of compound 27 bound to a BAZ2A chain and the indole ring of Trp1816 from a symmetric chain (Supplementary Fig. S2*a*). Different crystal polymorphs were observed in previous studies, induced by varying crystal contacts involving different BAZ2A inhibitors (PDB entries 7bl9, 7bla and 6fgg; Cazzanelli *et al.*, 2023 ▸; Dalle Vedove *et al.*, 2018 ▸).

**Figure 3:**
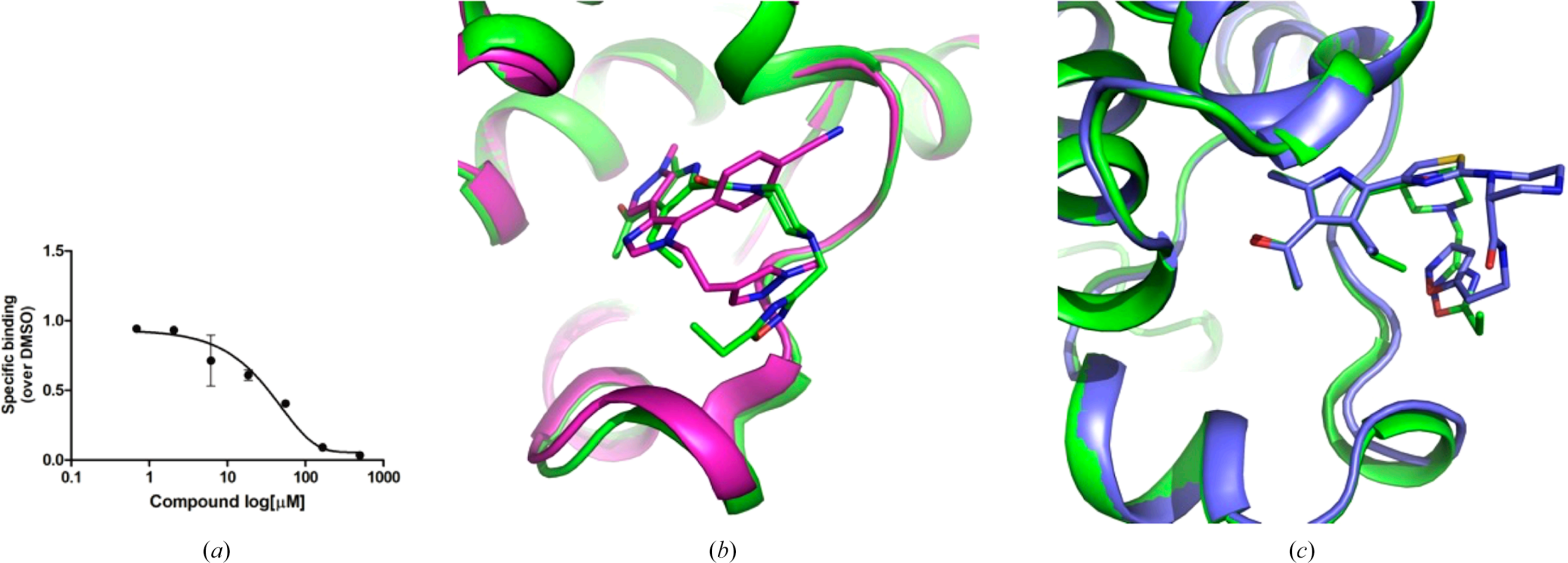
(*a*) Dose–response activity curve for compound **28** as measured in the AlphaScreen competition binding assay. (*b*) Superposition of compound **28** (green) and BAZ2-ICR (magenta). (*c*) Superposition of compound **28** (green) and an acetyl-pyrrole-thiazole compound with IC_50_ = 4.0 µ*M*.

Compound **28** reproduces all searched interactions with the charged piperazine in electrostatic contact with the Glu1820 side chain (3.5 and 3.7 Å from chains *A* and *B*, respectively), and the oxadiazole ring receiving a hydrogen bond from the Asn1823 main-chain nitrogen (Figs. 2 ▸*g* and 2 ▸*h*, Table 3 ▸ and Supplementary Fig. S3). The terminal ethyl group forms extensive contacts with Leu1826 and Val1827, at the same time being at a close distance to the ethyl substituent on the pyrrole ring (3.5 and 4.1 Å in chains *A* and *B*, respectively; Supplementary Fig. S3); this again suggests a macrocyclic evolution (Fig. 2 ▸*g*). Compound **28** performed better than all other compounds when tested in single dose (Table 2 ▸). Nonetheless, dose–response titration yielded an IC_50_ value of 34.2 ± 4.6 µ*M* (Fig. 3 ▸*a*), approximately an order of magnitude weaker than thiazole-pyrrole compounds that exploit similar interactions (Dalle Vedove *et al.*, 2022 ▸). It is conceivable that compound **28** pays both a conformational and an entropic penalty associated with its back-folded, almost enclosed, BAZ2A-binding pose.

Indeed, the oxadiazole ring in compound **28** occupies the same position as the oxazole ring of the most active thiazole-pyrrole compound. In the latter, however, the piperazine ring is derivatized in position 2, rather than position 4, favoring the backfolded organization, which is also stabilized by the stacking of the oxazole and thiazole rings (Fig. 3 ▸*b*). Interestingly, a very similar stacking interaction is observed in the most potent BAZ2A binder, the BAZ2-ICR chemical probe, suggesting an important compensatory role when constrained conformations are selected by the hydrogen bond with Asn1823 (Fig. 3 ▸*c*). Cyclization may represent an alternative strategy by predefining the required conformation rather than compensating for it. A conformationally restrained cyclic derivative of compound **28** may then improve BAZ2A binding, potentially reaching the single-digit micromolar range. A similar case has been previously reported for the BET bromodomain family, where cyclization drives an ∼40-fold increase in potency (Wang *et al.*, 2017 ▸). In a different case, cyclization was associated with both improved affinity and selectivity over the BET bromodomains (Jiang *et al.*, 2025 ▸). The BAZ2A complex with compound **28** crystallized in space group *P*2_1_2_1_2_1_, but with a different crystal packing than the complex with compound **27**; in this case, however, compound **28** is not involved in extended crystal contacts. It is conceivable that its binding rigidifies the ZA loop (amino acids 1820–1838) through the above-described interactions, allowing a tighter crystal packing (Supplementary Fig. S2*b*. Importantly, this excludes the backfolded conformation having been selected by the crystallization process.

In conclusion, we observed that compounds exploiting the most relevant interactions with BAZ2A in terms of both potency and specificity inspire the development of macrocyclic inhibitors. This strategy has already been successful for various drugs in clinical use (Zhang *et al.*, 2017 ▸).

## Supplementary Material

PDB reference: BAZ2A bromodomain, complex with compound **1**, 9f6w

PDB reference: complex with compound **23**, 9f70

PDB reference: complex with compound **26**, 9f71

PDB reference: complex with compound **27**, 9f77

PDB reference: complex with compound **28**, 9f78

Supplementary Figures. DOI: 10.1107/S2059798326004596/chr5011sup1.pdf

## Figures and Tables

**Table 1:** Data-collection and refinement statistics for BAZ2A structures Values in parentheses are for the outer shell.

	Compound **1**	Compound **23**	Compound **26**	Compound **27**	Compound **28**
Data collection					
Diffraction source	Elettra XRD2	Elettra XRD2	Elettra XRD2	Elettra XRD2	Elettra XRD2
Space group	*P*3_1_21	*P*3_1_21	*P*3_1_21	*P*2_1_2_1_2_1_	*P*2_1_2_1_2_1_
*a*, *b*, *c* (Å)	93.75, 93.75, 33.23	93.47, 93.47, 33.18	93.46, 93.46, 33.19	42.68, 55.02, 141.16	43.02, 54.36, 90.16
Wavelength (Å)	0.9997	0.9997	0.9997	0.9997	1.0000
Resolution (Å)	30.75–2.20 (2.27–2.20)	80.95–2.30 (2.38–2.30)	80.94–2.35 (2.43–2.35)	70.58–1.42 (1.44–1.42)	46.55–1.70 (1.73–1.70)
Total No. of reflections	163843 (12232)	143659 (13828)	126823 (7930)	787267 (37744)	127373 (6749)
No. of unique reflections	8667 (726)	7620 (759)	7143 (681)	63350 (3052)	22981 (1148)
*R*_merge_ (%)	19.8 (158.7)	36.5 (137.5)	10.4 (98.5)	9.5 (159.3)	7.1 (44.2)
*R*_meas_ (%)	20.3 (163.7)	37.6 (141.5)	10.7 (103.1)	9.9 (166.1)	7.9 (48.4)
*R*_p.i.m._ (%)	4.7 (39.6)	8.7 (33.0)	2.5 (29.8)	2.8 (46.5)	3.3 (19.2)
〈*I*/σ(*I*)〉	14.7 (2.4)	11.5 (2.9)	22.1 (2.5)	14.2 (2.0)	12.6 (3.0)
CC_1/2_	0.999 (0.846)	0.998 (0.912)	0.999 (0.813)	0.998 (0.692)	0.997 (0.924)
Completeness (%)	99.4 (98.6)	100.0 (100.0)	100.0 (100.0)	99.5 (99.6)	96.7 (93.7)
Multiplicity	18.9 (16.8)	18.9 (18.2)	17.8 (11.6)	12.4 (12.4)	5.5 (5.9)
Refinement					
Resolution (Å)	30.75–2.20	30.70–2.30	40.48–2.35	36.54–1.42	38.84–1.70
*R*_work_/*R*_free_ (%)	17.0/18.7	18.9/22.0	18.3/22.9	18.0/20.0	20.9/25.2
No. of non-H atoms					
Protein	866	875	888	2667	1739
Ligand	23	27	33	78	54
Water	55	67	42	392	256
Average *B* factors (Å^2^)					
Protein	49.5	39.8	56.2	26.7	31.8
Ligand	44.0	34.8	57.3	26.7	25.0
Water	45.8	37.8	51.0	36.2	35.4
R.m.s. deviations					
Bond lengths (Å)	0.006	0.007	0.004	0.005	0.005
Bond angles (°)	0.791	0.927	0.719	0.882	0.833
Ramachandran plot					
Most favored (%)	100	98.02	97.03	100	99.0
Allowed (%)	0	1.98	2.97	0	1.0
PDB entry	9f6w	9f70	9f71	9f77	9f78

**Table 2:** Structures and activities of tested compounds

Compound	Structure	Residual BAZ2A binding at 100 µ*M* (%)	Compound	Structure	Residual BAZ2A binding at 100 µ*M* (%)
**1**	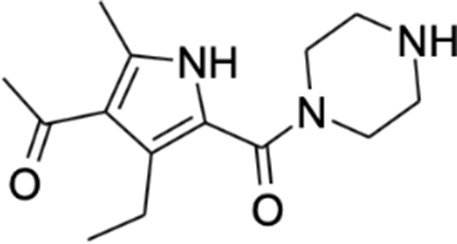	64 ± 1	**15**	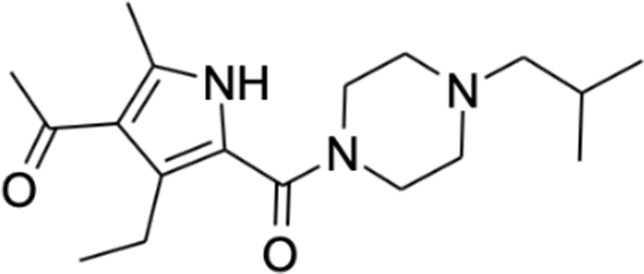	48 ± 6
**2**	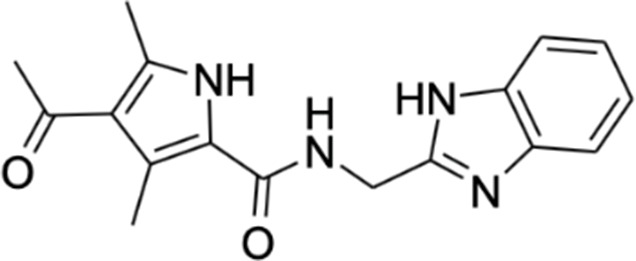	82 ± 3	**16**	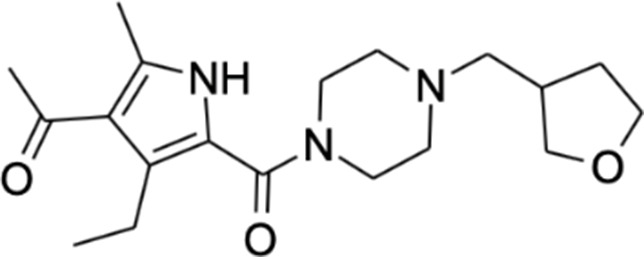	43 ± 10
**3**	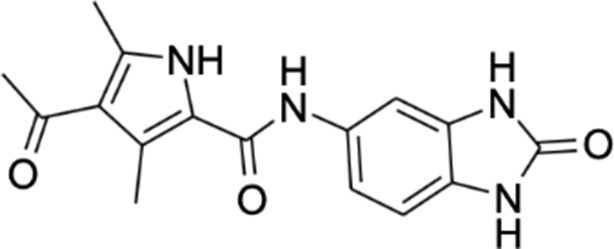	82 ± 1	**17**	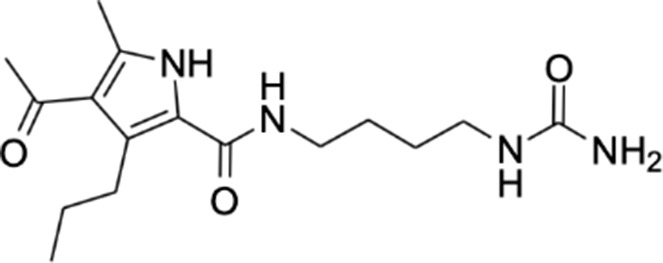	83 ± 14
**4**	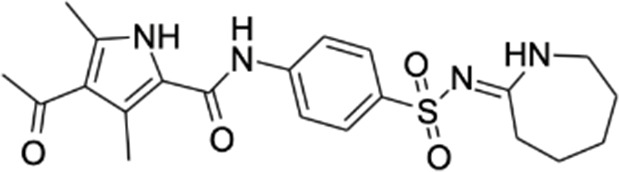	84 ± 4	**18**	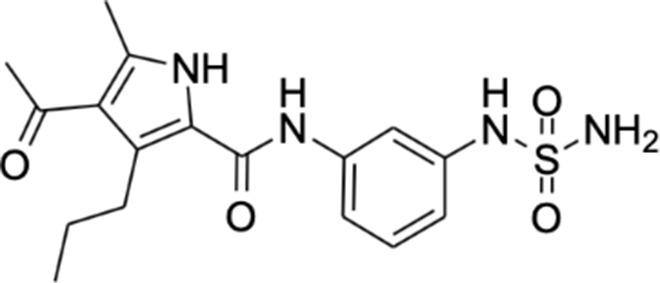	100 ± 6
**5**	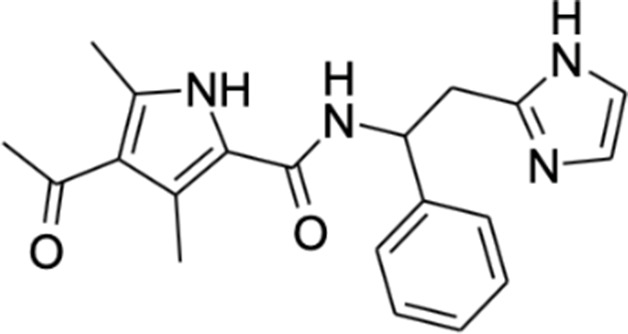	54 ± 3	**19**	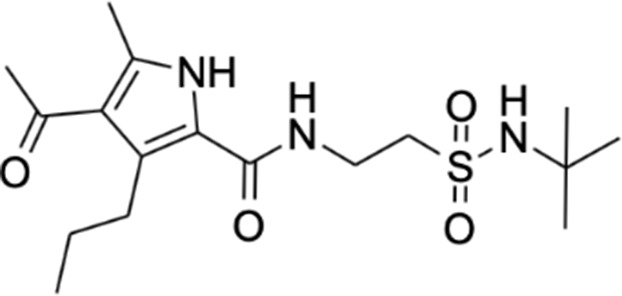	100 ± 7
**6**	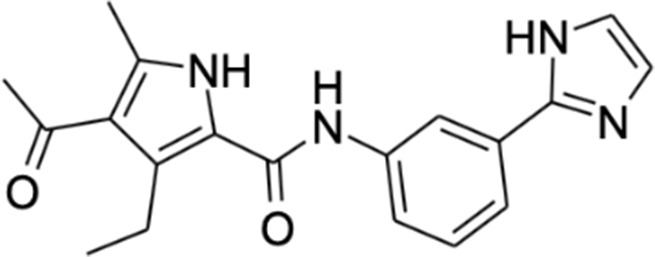	97 ± 6	**20**	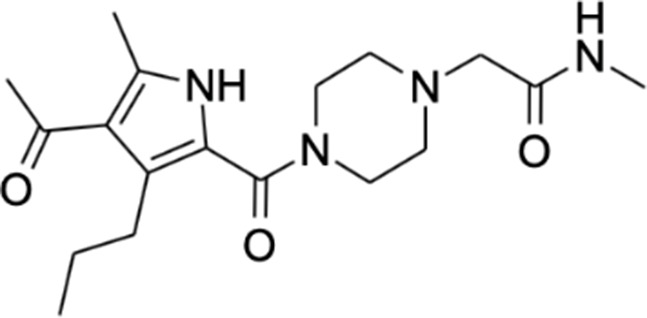	91 ± 5
**7**	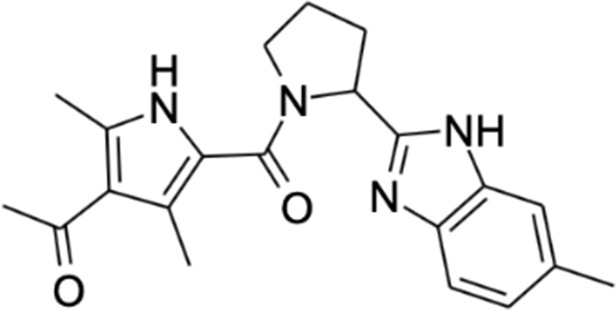	72 ± 4	**21**	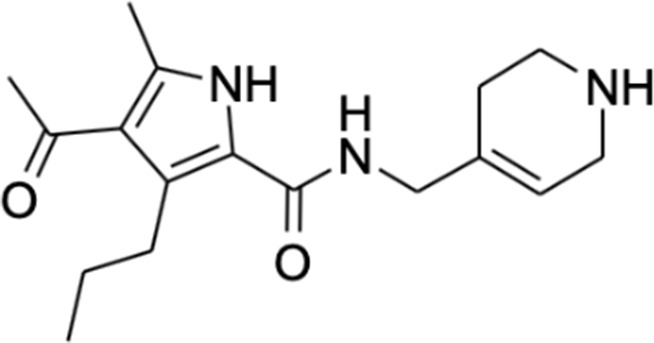	66 ± 11
**8**	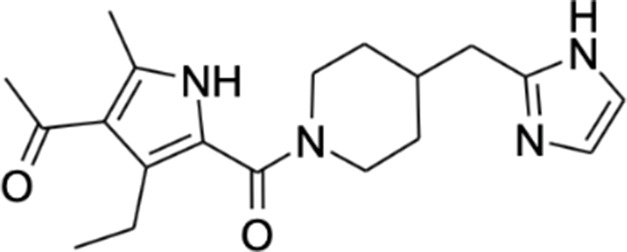	78 ± 9	**22**	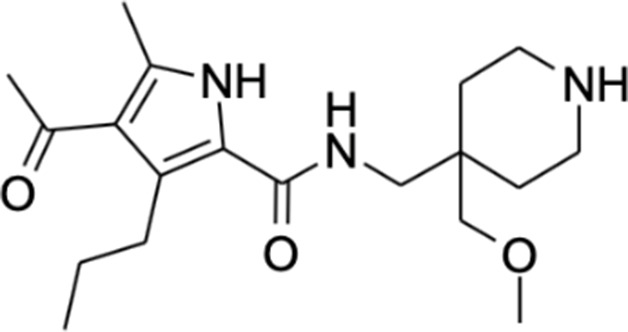	55 ± 1
**9**	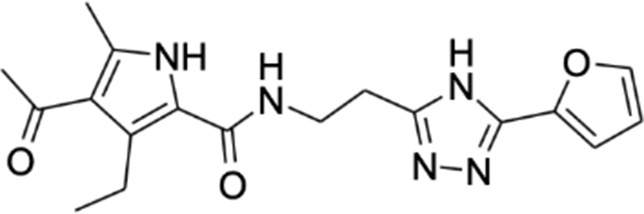	96 ± 12	**23**	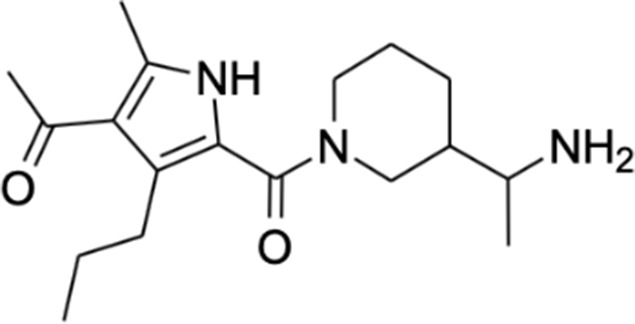	49 ± 1
**10**	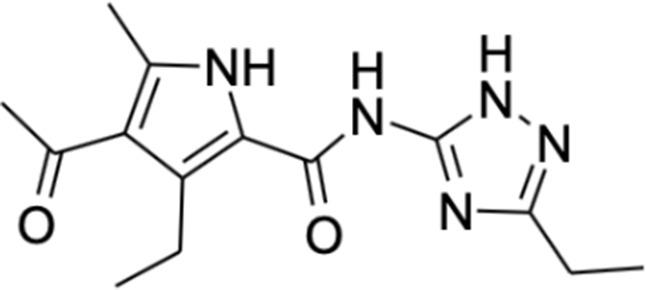	100 ± 9	**24**	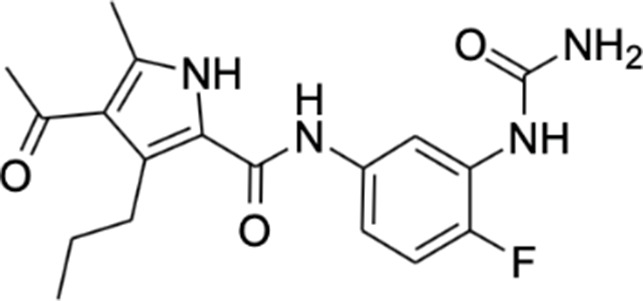	100 ± 7
**11**	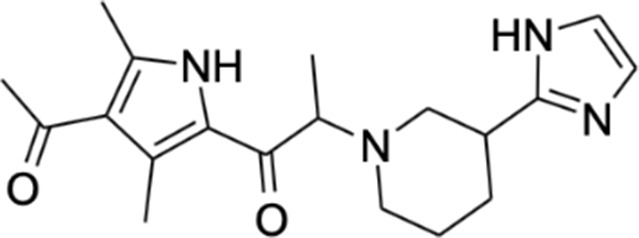	68 ± 4	**25**	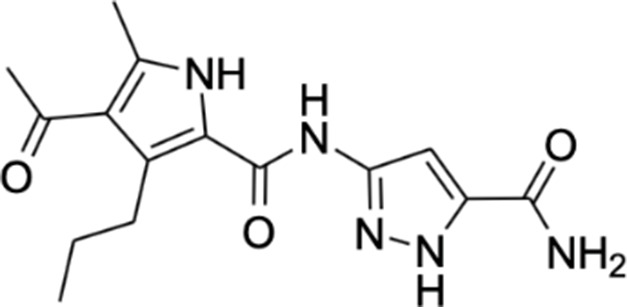	44 ± 5
**12**	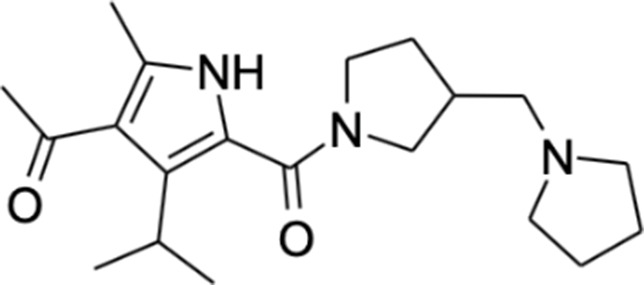	78 ± 6	**26**	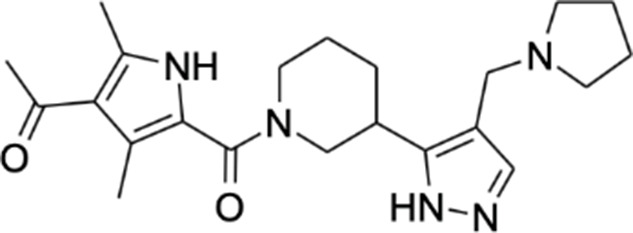	45 ± 4
**13**	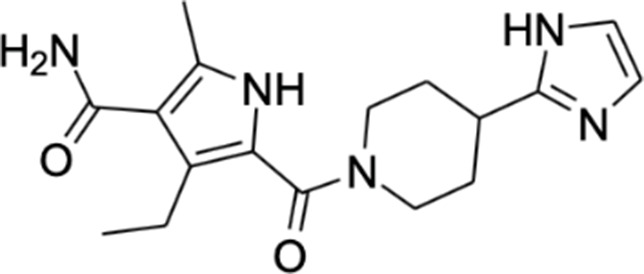	81 ± 9	**27**	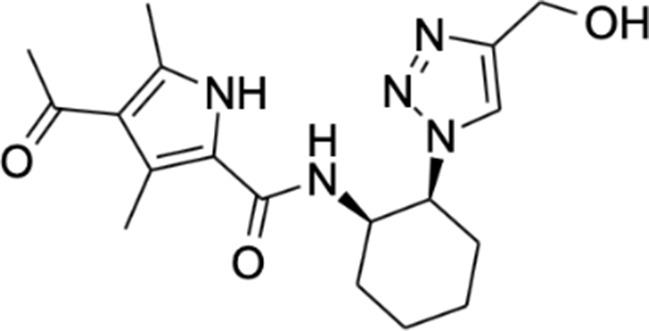	49 ± 5
**14**	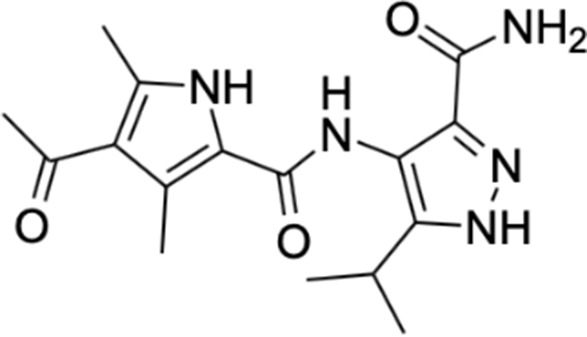	80 ± 1	**28**	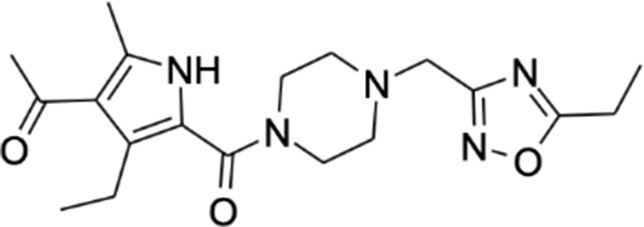	27 ± 8

**Table 3:** Interaction distances and areas for the crystallized complexes Interactions are reported for distances of ≤4.5 Å. Hydrogen bonds are shown in bold and ionic interactions in bold italic.

				Compound **27**			Compound **28**	
Residue	Compound **1**	Compound **23**†	Compound **26**†	Chain *A*	Chain *B*	Chain *C*	Chain *A*	Chain *B*
Minimal distance (Å)/residue buried surface area (Å^2^)								
Trp1816	4.1/28.0	4.0/36.2	3.5/42.4	4.4/17.5	3.6/26.3	4.1/26.8	3.9/22.1	4.2/21.4
Pro1817	**2.9/22.4**	**2.8/23.4**	**2.7/24.1**	**2.8/20.8**	**2.8/21.9**	**2.9/23.2**	**2.7/24.5**	**2.8/23.6**
Phe1818	3.9/9.0	3.6/10.4	4.0/9.6	3.8/10.3	3.7/8.9	3.8/9.5	3.9/8.9	3.9/8.0
Glu1820	***3.1/23.5***	***3.5/43.1***	3.4/43.7	3.8/19.9	4.0/17.8	3.9/20.3	***3.3/40.0***	***3.3/39.7***
Pro1821	3.7/6.2	3.9/4.0	4.3/2.9	3.3/9.8	3.2/10.5	3.6/8.5	3.5/5.1	3.5/4.5
Val1822	3.5/39.1	3.4/38.3	3.4/38.0	3.5/36.5	3.6/37.4	3.5/37.5	3.6/40.8	3.6/42.1
Asn1823	—	—	—	**2.9/20.9**	**3.1/16.0**	**2.9/16.9**	**3.0/11.1**	**3.1/13.0**
Leu1826	—	—	—	3.7/33.4	3.3/30.9	3.7/36.0	3.6/30.9	3.5/33.4
Val1827	3.9/22.3	3.9/29.3	3.6/19.0	3.9/35.0	3.5/32.1	3.8/30.9	3.8/30.8	3.7/29.1
Tyr1830	3.8/10.1	3.6/11.1	3.7/11.2	3.7/11.7	3.6/10.2	3.7/11.1	3.8/9.4	3.7/9.1
Phe1872	3.4/5.0	3.4/6.4	3.7/5.2	3.5/3.7	3.4/3.6	3.6/3.9	3.5/7.0	3.4/7.7
Asn1873	**2.7/18.4**	**2.7/18.9**	**2.8/13.9**	**2.8/16.2**	**2.8/14.0**	**2.9/15.8**	**2.9/13.7**	**3.0/13.7**
Val1879	3.7/36.6	3.7/34.9	3.7/32.2	3.8/31.3	3.8/30.3	3.8/32.5	3.8/28.6	3.7/28.6
Overall buried surface area (Å^2^)								
BAZ2A	220.6	256.1	242.2	267.0	259.9	273.0	273.1	273.8
Compound	331.6	355.2	337.6	395.9	392.9	390.7	412.0	408.0

† All stereoisomers are considered.
