# Supplementary material for: Unusual backfolded binding poses of BAZ2A bromodomain binders
Source: Acta Crystallogr D Struct Biol. 2026 Jun 3;82(Pt 7):776–84. doi: 10.1107/S2059798326004596 (PMC13317689; doi:10.1107/S2059798326004596)
Supplement: Supplementary file 1 [file d-82-00776-sup1.pdf]

STRUCTURAL BIOLOGY

Volume 82 (2026)

Supporting information for article:

Unusual backfolded binding poses of BAZ2A bromodomain binders

Andrea Dalle Vedove, Giulia Cazzanelli, Vito Giuseppe D'Agostino, Pablo Andres Vargas Rosales, Amedeo Caflisch and Graziano Lolli

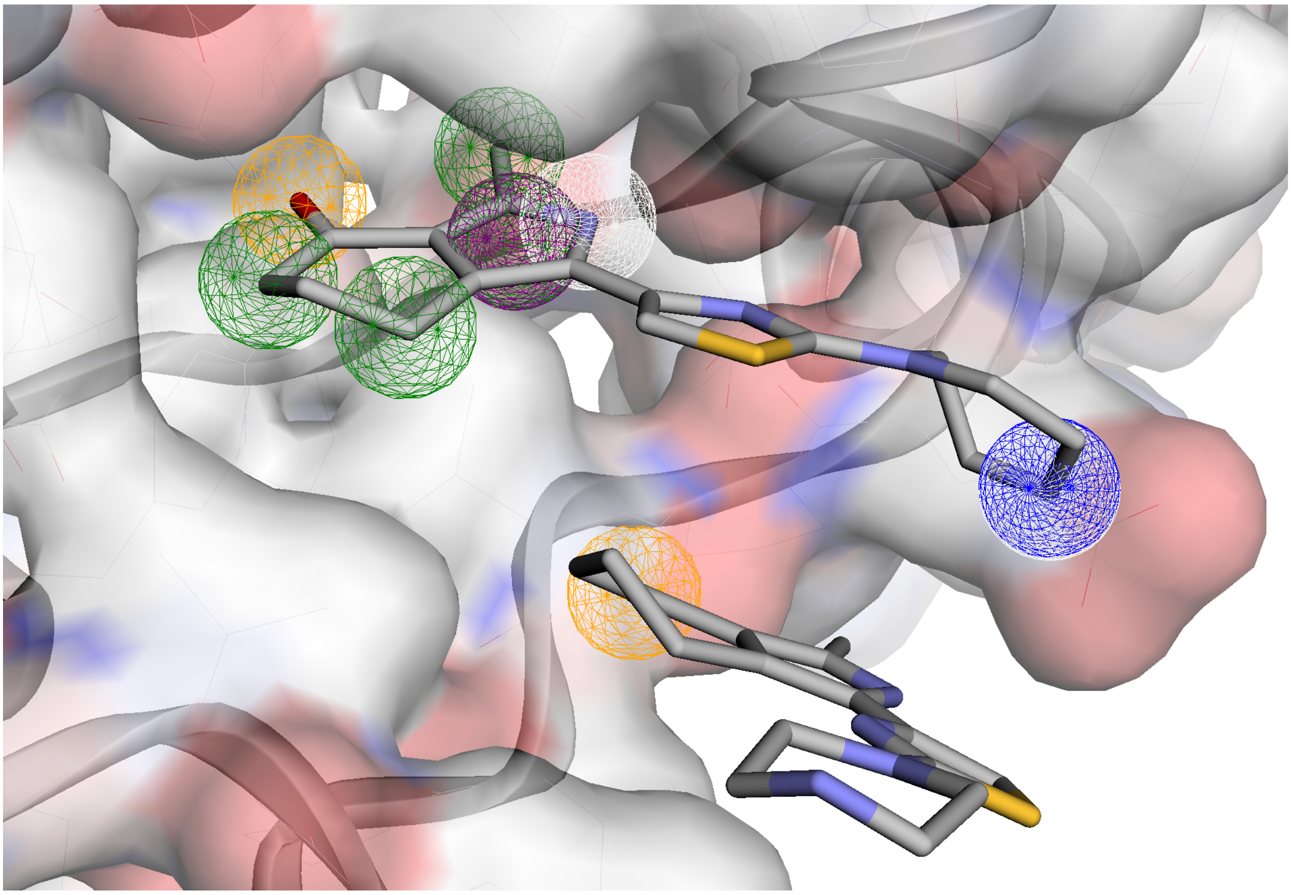

Supplementary Figure S1. Pharmit search. PDB 7QYT was used for the pharmacophore search. Orange spheres: H-bond acceptor for interaction with Asn1823 and Asn1873. White spheres: H-bond donor for interaction with Pro1817 and Glu1820. Blue sphere: positive charge for interaction with Glu1820. Green spheres (hydrophobic) and purple sphere (aromatic) for conserved interactions at the bottom of the pocket.

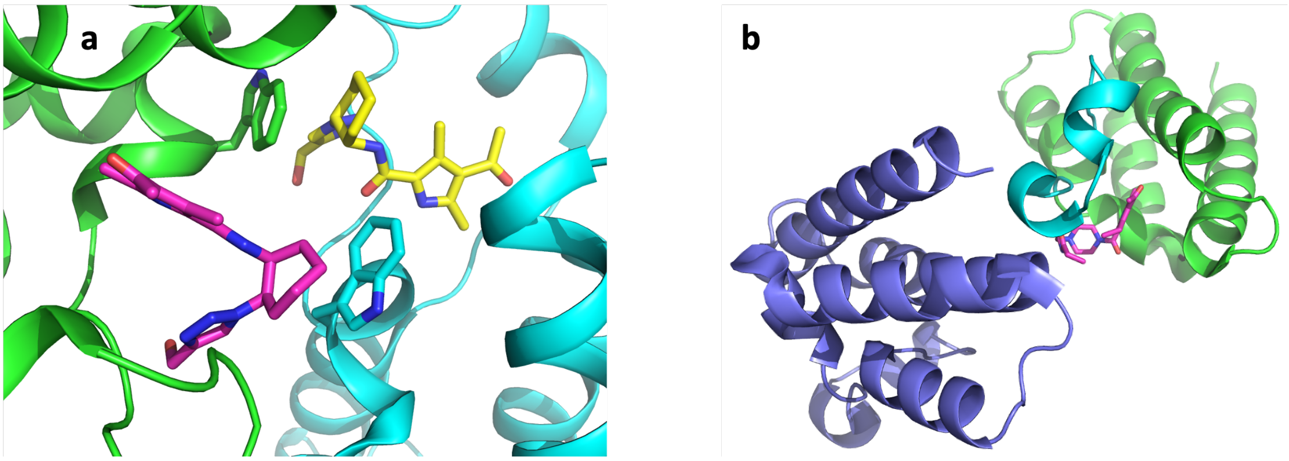

Supplementary Figure S2. Crystallographic packings. a) Compound **27** favors the crystallographic packing by staking to W1816 of a symmetric chain. b) Compound **28** is not involved in extensive crystallographic contacts; packing may be ascribed to interactions with symmetric chains of the ZA loop (cyan) rigidified by compound **28** binding.

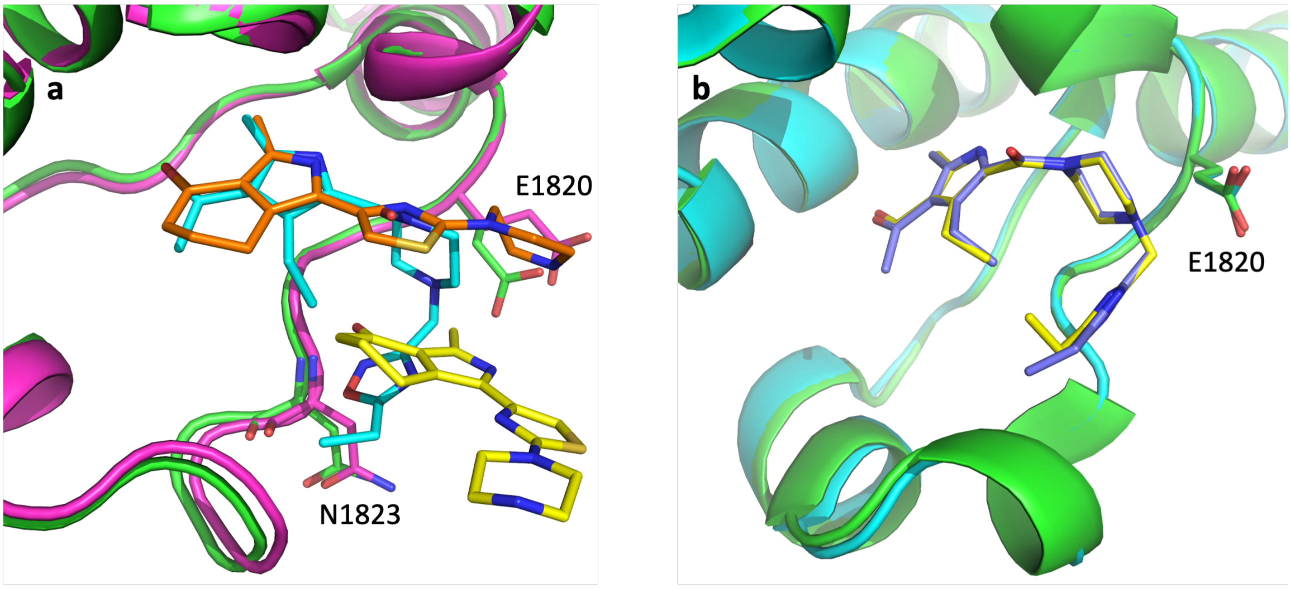

Supplementary Figure S3. a) Compound **28** (cyan, BAZ2A shown in green) exploit all interactions selected in the Pharmit search by using a prototype compound (PDB 7QYT, orange and yellow, BAZ2A shown in magenta). b) Compound **28** assumes very similar poses in the two chains of the asymmetric unit.
